# The non-uniformity correction factor for the cylindrical ionization chambers in dosimetry of an HDR ^192^Ir brachytherapy source

**DOI:** 10.4103/0971-6203.26893

**Published:** 2006

**Authors:** Bishnu Majumdar, Narayan Prasad Patel, V. Vijayan

**Affiliations:** Department of Physics, Govt. College of Science, Raipur, India; * Department of Medical Physics, Acharya Harihar Regional Cancer Centre, Cuttack, India; ** Department of Health Physics Unit, Institute of Physics, Bhubaneswar, India

**Keywords:** Brachytherapy source, dosimetry, ionization chamber, non-uniformity correction factor

## Abstract

The aim of this study is to derive the non-uniformity correction factor for the two therapy ionization chambers for the dose measurement near the brachytherapy source. The two ionization chambers of 0.6 cc and 0.1 cc volume were used. The measurement in air was performed for distances between 0.8 cm and 20 cm from the source in specially designed measurement jig. The non-uniformity correction factors were derived from the measured values. The experimentally derived factors were compared with the theoretically calculated non-uniformity correction factors and a close agreement was found between these two studies. The experimentally derived non-uniformity correction factor supports the anisotropic theory.

The dosimetry in the brachytherapy plays a vital role to ensure an accurate dose delivery to the target volume. The ICRU report 24 recommends the delivered dose to the target volume must be within ±5% of the prescribed dose.[1] The AAPM report recommends several factors for the dose calculation at any point, which are air kerma strength, dose rate constant, radial dose function and anisotropy function. The uncertainty in determination of each factor is about ±5%. However, adding these uncertainties in quadrature, the overall uncertainty in determination of dose rate at a point around a source using the recommended protocol is estimated to be about ±10%.[2] Several dosimetry systems are being used for the dose measurement of the brachytherapy sources. The criteria for the selection of dosimetry system are the type and energy of radiation, availability, sensitivity, stability, energy dependence, environmental conditions, size of the detector and the cost. Each of the dosimetry systems has its own advantages and disadvantages for the dose measurement of the brachytherapy sources. The ionization chamber is energy-independent and shows linear response to the dose; however, the steep dose gradient inside the active volume nearer to the brachytherapy source gives large uncertainties in the measurement. The ionization chambers are commonly available in the radiotherapy centers for the dosimetry of the external beam therapy.

The non-uniformity correction factor accounts for the non-uniform photon fluence over the ionization chamber caused by the divergence of the incident photons. The effect due to divergence of photons is greatest for ionization chamber dosimetry in the vicinity of brachytherapy sources. A number of theories have been proposed for the determination of the non-uniformity correction factors. Mayneord and Roberts performed an integration of the inverse square law over the air cavity of the ionization chamber.[3] Spiers pointed out that more accurate value of the non-uniformity correction factors should be obtained if the integration were restricted to the inner wall of the ionization chamber.[4] Kondo and Randolph proposed an electron transport model assuming that the angular distribution of the electrons entering the cavity is isotropic.[5] The theory given by Kondo and Randolph was later extended by Bielajew, having a more realistic anisotropic angular distribution of the electron fluence within the air cavity of the ionization chamber.[6] Monte Carlo calculation by Bielajew later gave substantial support to the anisotropic theory.[7]

Tölli *et al*. performed an experimental study on the non-uniformity correction factor using various chambers of different radii and materials of the central electrode.[8] Their study supported the anisotropic behavior proposed by Bielajew at short distances. They also concluded that the choice of material and diameter of central electrode do not contribute significantly to the chamber's non-uniformity correction factors. An error in the equations given by Kondo and Randolph was discovered during the course of their study and the correct version of equation was reported in the publication.

The aim of the present study is to determine the non-uniformity correction factor for the 0.1 cc and 0.6 cc ionization chambers with high dose rate ^192^Ir brachytherapy source. The non-uniformity correction factors are derived from in-air measurement for shorter distances between the source and chamber. The experimentally derived factors are compared with the theoretically calculated non-uniformity correction factors.

## Materials and Methods

### A literature review of the non-uniformity correction factor

The incident photons with high divergence and the non collimated geometry in the measurements of brachytherapy sources, free in-air or in any other medium, differ from the geometry of the collimated photon beams such as those external beams used for calibrating the chamber. There will be a marked variation in the photon fluence over the different parts of sensitive volume of the chamber. The electrons entering the air cavity are mainly generated in the inner wall of the chamber. Due to the non-uniform photon fluence over the wall, the generation of electrons from the wall varies significantly from place to place in the wall. The net result is non-uniform electron fluence in the air cavity of the chamber. This non-uniformity is very much significant even for the small detectors at shorter distances from the brachytherapy sources. In order to take into account this non-uniformity so as to convert the measured charge or current into air kerma rate at the measurement distance, it is necessary to apply the non-uniformity correction factor. The following factors influence the non-uniformity correction factor (NUCF):

- The non-uniformity correction factor decreases with the increase in distance between the source and the ionization chamber. For the larger distances, NUCF approaches unity asymptotically.
- In measurements made in air with cylindrical ionization chamber near point sources, the dependence on the geometry is well described by the ratio of the chamber diameter to the length of the chamber.[5]
- Different source geometries yield different gradients and therefore different non-uniformity correction factors. In a special case, when attenuation and scattering from air is neglected, the kerma in air, K, is proportional to 1/r_0_^2^, where r_0_ is the distance from a point source to the point of interest. In line source dosimetry, when the filtration within the source and the capsule is neglected, the kerma along a line through the center of the line source and the perpendicular to the source axis is proportional to (2/Lr_0_) tan^-1^ (L/2r_0_),[9] where *r*_0_ is the distance from the center of the line source to the point of interest and L is the active length of the source.
- Different primary photon energies yield different energy and angular distributions of the scattered photons and therefore different gradients. The non-uniformity correction factor therefore depends on the initial energy of the photons and hence the non-uniformity effect in a solid phantom is different from that in air. Furthermore, the number of scattered photons increases with the distance from the source and the non-uniformity effect should therefore vary with the distance due to scattering. However, the non-uniformity of the fluence within the cavity is mainly caused by the inverse square law, which is independent of the energy of the photons and the medium properties and therefore, the dependence on the energy is expected to be small.

**The theoretical non-uniformity correction factor:** Kondo and Randolph took up the suggestion given by Spiers[4] for a surface integral theory as almost all the ionization produced in properly designed chambers exposed to high energy photons is generated by secondary electrons originating in the internal surface of the chamber wall. The non-uniformity correction factors given by Kondo and Randolph[5] are most widely used. In their theory, the electron fluence in the air cavity of the ionization chamber is assumed to be isotropic. The theory was later extended by Bielajew,[6] who included a more realistic angular distribution of electron fluence in the air cavity of the chamber. In contrast to the isotropic theory, this anisotropic theory predicts the wall material and a photon energy dependence in the non-uniformity correction factor. The relationship between the two theories is given by

(1) $$A_{pn}(r)=A_{pn}^{KR}(r)+\omega A^{'}(r)$$

Where 1/ *A^KR^(r)* is the non-uniformity correction factor from the isotropic theory of Kondo and Randolph[5] and 1/*A_pn_(r)* is the non-uniformity correction factor according to the theory of Bielajew.[6] The factor *A′_pn_(r)* takes into account the anisotropic electron fluence within the air cavity and the degree of anisotropy is given by the energy and material dependence factor ω.

It is recommended in the report[10] that the factor 1/*A_pn_(r)* according to the theory by Bielajew be used for determination of non-uniformity correction factor (*K_n_*). Thus,

(2) $$K_{n}\;=\;1/A_{\mathrm{pn}}\left(r\right)$$

The parameters ${A_{\mathrm{pn}}}^{\mathrm{KR}}\left(r\right)$ *(r)* and *A′_pn_(r)* for the calculation of the non-uniformity correction factor for cylindrical chambers are given in literature.[8] These parameters are given in table as a function of the cylindrical chamber's shape factor, σ = *R_c_* / *L_c_* and the distance factor, α = *R_c_* / *r*, where *R_c_* is the chamber's internal radius and *L_c_* is the internal half-length of the chamber and *r* is the measurement distance.

**An experimental non-uniformity correction factor:** It is well known that the primary radiation from point source obeys the inverse square law. However, this inverse square law does not apply on the measured values by the finite size ionization chamber at shorter distances from the source. Neglecting the attenuation and scattering in air medium and the variation of inverse square law over the ionization chamber at distances, the measured value *M*_u_(*r*_0_), at a distance *r*_0_ between a point source and the center of the ionization chamber, can be expressed as[11]

(3) $$M_{u}(r_{0})=\frac{c}{r_{0}^{2}}\;\mathrm{(3)}$$

Where *c* is constant of proportionality. The ratio of measured values at two different distances, *r*_1_ and *r*_2_, is therefore

(4) $$\frac{M_{u}(r_{1})}{M_{u}(r_{2})}={\left(\frac{r_{2}}{r_{1}}\right)}^{2}\;\mathrm{(4)}$$

At short distances, the inverse square law variation over the ionization chamber can no longer be neglected and the validity of Equation (4) no longer exists. In order to obtain the inverse square relation, the measured value must be corrected with the non-uniformity correction factor *P_n_*, yielding

(5) Mu(r1)Pn(r1)Mu(r2)Pn(r2)=(r2r1)2(5)

or

(6) $$\frac{P_{n}(r_{1})}{P_{n}(r_{2})}={\left(\frac{r_{2}}{r_{1}}\right)}^{2}\left(\frac{M_{u}(r_{2})}{M_{u}(r_{1})}\right)\;\mathrm{(6)}$$

Where *r*_1_ and *r*_2_ are two different distances between point source and the center of the ionization chamber. From Equation (6), ratios of non-uniformity correction factors can be determined. As the measurement distance is increased, the inverse square law variation across the cavity is reduced and the non-uniformity correction factor approaches unity asymptotically.

If the distance *r*_2_ is selected to be large enough, then *P_n_*(*r_2_*) = 1 and the absolute values of the *P_n_* factors can be derived. Thus, near the point sources,

(7) $$P_{n}(r_{1})={\left(\frac{r_{2}}{r_{1}}\right)}^{2}\left(\frac{M_{u}(r_{2})}{M_{u}(r_{1})}\right)\;\mathrm{(7)}$$

provided that *r*_2_ is selected at large distance, where the size of the ionization chamber can be neglected.

When the line sources are used in the measurements and with the same assumptions as those yielding Equation (3),

(8) $$M_{u}(r_{0})=\left[\frac{2cG_{F}(r_{0})}{L_{s}r_{0}}\right]\tan^{-1}\left(\frac{L_{s}}{2r_{0}}\right)\;\mathrm{(8)}$$

where *L_S_* is the active length of the source, *r*_0_ is the distance from the center of the line source to the ionization chamber and *c* is the constant of proportionality. The filtration factor *G_F_*(*r_0_*) in Equation (8) takes into account the gradient caused by the filtration within the source and the capsule. For unfiltered line sources, *G_F_*(*r_0_*) = 1. With the two different measurement distances, *r*_1_ and *r*_2_, the ratio becomes

(9) $$\frac{M_{u}\left(r_{1}\right)}{M_{u}\left(r_{2}\right)}=\frac{r_{1}G_{F}\left(r_{1}\right)\tan^{-1}\left(\frac{L_{s}}{2r_{1}}\right)}{r_{1}G_{F}\left(r_{2}\right)\tan^{-1}\left(\frac{L_{s}}{2r_{2}}\right)}\;\mathrm{(9)}$$

When the electron fluence variation over the chamber cannot be neglected, the equation analogous to Equation (5) is

(10) $$\frac{M_{u}\left(r_{1}\right)P_{n}\left(r_{1}\right)}{M_{u}\left(r_{2}\right)P_{n}\left(r_{2}\right)}=\frac{r_{2}G_{F}\left(r_{1}\right)\tan^{-1}\left(\frac{L_{s}}{2r_{1}}\right)}{r_{1}G_{F}\left(r_{2}\right)\tan^{-1}\left(\frac{L_{s}}{2r_{2}}\right)}\;\mathrm{(10)}$$

By the same argument as earlier, *r*_2_ is selected at large distance where the size of the ionization chamber can be neglected, so that *P_n_*(*r*_2_) = 1 and absolute values of the factors are obtained from

(11) $$P_{n}r_{1}=\frac{M_{u}\left(r_{1}\right)r_{2}G_{F}\left(r_{1}\right)\tan^{-1}\left(\frac{L_{s}}{2r_{1}}\right)}{M_{u}\left(r_{1}\right)r_{1}G_{F}\left(r_{2}\right)\tan^{-1}\left(\frac{L_{s}}{2r_{2}}\right)}\;\mathrm{(11)}$$

In the above equation, the non-uniformity correction factor is derived from measured value using any chamber with the line source of active length *L_S_*. It should be noted that the factors derived from above equation are applicable only for measurements using pair of similar chamber and line source.

### An experimental procedure

The non-uniformity correction factors for two small cylindrical ionization chambers were derived by experimental procedure. The 0.6 cc Farmer-type and 0.1 cc *in-vivo* type ionization chambers from PTW, Freiburg, Germany, were used. The internal length and the diameter of the ionization chambers were 1.3 and 0.35 cm respectively for the 0.1 cc chamber and similarly, 2.1 and 0.61 cm for the 0.6 cc chamber. The material used in the inner wall of both the chambers was PMMA. The buildup cap was used for the 0.6 cc ionization chamber in measurement; whereas the 0.1 cc chamber did not have any buildup cap.

**Figure 1:**
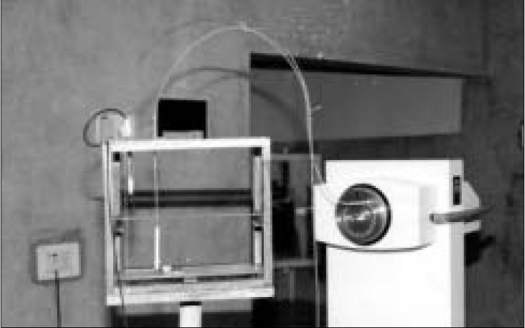
Measurement jig with 0.1 cc chamber and brachytherapy unit

Figure 1 shows the specially designed measurement jig. The dimensions of the jig were about 30 × 20 × 27 cm. It was made of low Z materials of acrylic plates and wooden frames to have minimum contribution from the scattered radiation. There are two scale systems in the measurement jig – one on the top and the other on the midplane of the jig. Two scale systems were aligned parallel with the help of laser beams. The scale systems were used to determine the measurement distance and also for vertical positioning of the source applicator. The chamber holder can be mechanically fixed at one position in the jig, whereas the source applicator holder was shifted linearly along the track. The fine laser beam was projected over the jig for verification of the sagittal, transverse and coronal cross-sectional planes. The source applicator (inner and outer diameter of 1.35 and 1.65 mm respectively) can be placed at any scale pointer with an accuracy of about ± 0.01 cm.

First, the source applicator was placed at 1 cm distance from the chamber. The successive measurements were performed by vertical scanning of the source to determine the reference dwell position where chamber shows maximum response. In the vertical scanning, the source was moved to dwell positions (stepping distance of 2 mm) along the applicator. The reference dwell position where the chamber shows maximum response lies on the transverse axis that passes through the center of the source as well as the chamber. For measurement of air kerma, the source applicator was placed at measurement distance and source was moved to the reference dwell position. The air kerma were measured for the different source-to-chamber distances. The source-to-chamber distances varied from 1 to 20 cm for the 0.6 cc chamber, whereas for the 0.1 cc chamber, this range was 0.8 to 16 cm. The period of air kerma measurement varied from 30 s to 200 s, depending upon the measurement distance, size of the chamber and activity of the source. The measured values were corrected for time factor and the air attenuation.[10] Transit time correction was not required as dosimeter timer was used to collect the charge during an interval when the source was stopped. The leakage current was insignificant and change in temperature and pressure was monitored for the correction.

The air kerma readings taken at different measurement distances (more than five) for more than 10 cm were corrected for the non-uniformity correction factor.[8,10] The measured air kerma *M_d_* has contribution from the scattered radiation *M_S_*. The correction for the room scatter[12] was made in order to know the measured air kerma reading from the primary radiation, i.e., *M_u_* = *M_d_* - *M_S_*. The products of air kerma *M_u_* and square of the distance were calculated. The mean of these products from different measurement distances was taken as reference value. This value was considered as product of measured air kerma at infinite distance where the non-uniformity correction factor due to chamber size was absolute unity. The non-uniformity correction factors were then calculated for various distances between 0.8 and 10 cm from the source, using Equation (11).

## Results and Discussion

The minimum possible measurement distance for the 0.1 and 0.6 cc ionization chambers in our setup was 0.8 and 1.0 cm respectively. 0 The maximum response of the 0.6 and 0.1 cc chambers were found with the source stopping position at 6.8 and 5.4 cm from the end of the source applicator respectively. This means that the heights of the effective centers of the chambers from the base of the jig differed by 1.4 cm. Reproducibility (n = 5) of our measurement by repositioning of the source applicator was within 3, 1 and 0.1% at measurement distances of 1, 5 and 10 cm respectively. Overall, the standard deviation in measured values *M_u_r^2^* (for *r* > 10 cm) was found to be well within 0.2 and 0.3% of the mean for the 0.6 and 0.1 cc chambers respectively. The scattered radiation M_S_ measured for 0.1 and 0.6 cc chambers was 1.1 and 0.33% of the primary radiation at 10 cm measurement distance from the source respectively.[12]

The GammaMed Plus high dose rate ^192^Ir source was used in the present study. The active length of the source was 0.35 cm with stainless steel (encapsulated) of thickness 0.015 mm. The effect of the encapsulation on the non-uniformity correction factor was assumed to be insignificant. The study of non-uniformity correction factor based on the inverse square law by Patel *et al.*[13] shows that the effect of the filtration from source encapsulation on the correction factor was 0.01% at 0.4 cm measurement distance for the 0.1 cc chamber. Due to this very small effect, the thickness of the source filter was not considered in the calculation using Equation (11) and the value of factors *G_F_*(*r*_1_) and *G_F_*(*r*_2_) was taken one.

The theoretical values of NUCF were estimated on the assumption of the point source. However, the high dose rate ^192^Ir source used in this experimental study was line source of length of 0.35 cm. Tölli *et al.*[8] derived a formula to average the theoretical values of point source formalism (*A_pn_*(*r*)) over the length of the source. They found that the averaging increased the theoretical values by approximately 0.5 and 0.3% relative to point-source formalism at a distance of 0.8 and 1.0 cm respectively. This means the non-uniformity correction factors decrease for the line source as *K_n_* = 1/*A_pn_*(*r*). At larger distances, this difference decreases and the line source can be assumed to be a point source. The inner wall of the chamber was made up of PMMA. In the theoretical calculation, the value of ω used for both the chambers was 1.0 14.[8] The shape factor σ for the 0.1 and 0.6 cc chambers was 0.291 and 0.265 respectively.

The non-uniformity correction factor for the 0.1 and 0.6 cc chambers from experimental and theoretical studies is shown in Table 1. The measurement distance covered by the 0.1 cc chamber varies from 0.8 to 8.0 cm, whereas for the 0.6 cc chamber, it is 1.0 to 10 cm. The comparison between the measured and theoretical values of non-uniformity correction factor in Table 1 shows good agreement. Although the theoretical values are little higher than the measured values at shorter distance, when these values will be averaged for the line source, there will be very good agreement between theoretical and experimental values. There might be some variation in the non-uniformity correction factor as interpolation method was used to derive the factor *A_pn_*(*r*) in the theoretical calculation. The experimental values of the non-uniformity correction factor for 0.6 cc chamber at distance of 1, 2 and 10 cm was found to be 1.3207, 1.116 and 1.011 respectively, as against the theoretical values of 1.3333, 1.1196 and 1.0064 respectively. Generally the volume of the 0.1 cc chamber is considered to be very small and the non-uniformity correction factor is not taken into consideration for the dose measurement near the brachytherapy source. In our study, the non-uniformity correction factor for 0.1 cc chamber was found to be 1.107, 1.033 and 1.006 at a distance of 1, 2 and 5 cm from the source. Our present study shows that the non-uniformity correction factor supports the anisotropic theory. As the studies by Tölli *et al*.[8] have shown, at shorter distance the agreement with the isotropic behavior was about 3%, whereas with the anisotropic theory, it was 1%.

**Table 1:** Non-uniformity correction factor of the 0.1 and 0.6 cc ionization chamber for distances from 0.8 to 10.0 cm from theoretical and experimental study. Theoretical values were calculated by extrapolation method for the point source, whereas the measured values were for 0.35 cm length source

*Distance (cm)*	*Non–uniformity correction factor*			

		*0.1 cc chamber*		*0.6 cc chamber*	
		
	*Experimental*	*Theoretical*	*Experimental*	*Theoretical*
0.8	1.166	1.175	-	-
1.0	1.107	1.126	1.3207	1.3333
1.2	1.08	1.098	1.2403	1.2517
1.5	1.06	1.0681	1.176	1.1795
1.8	1.058	1.0495	1.123	1.1451
2.0	1.033	1.0422	1.116	1.1196
2.5	1.024	1.0304	1.0813	1.0836
3	1.019	1.022	1.0628	1.0595
4.0	1.012	1.0126	1.0404	1.0398
5.0	1.006	1.0099	1.033	1.0279
6.0	1.0045	1.0078	1.0248	1.0198
7.0	-	-	1.0131	1.0133
8.0	1.0044	1.005	1.0185	1.0138
10.0	-	-	1.011	1.0064

**Figure 2:**
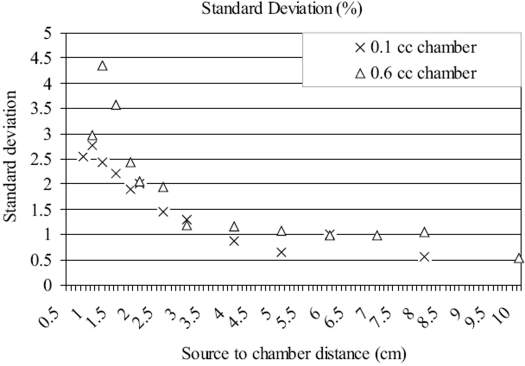
Standard error *vs*. distance between source and chamber in the measurement of non-uniformity correction factor for 0.1 and 0.6 cc chambers (N = 10 to 25)

The positioning error was the major source of uncertainty in our measurement, which follows the inverse square law – increasing with decrease in the source-to-chamber distance. An accuracy of positioning of canter of the chamber was best known to be ±0.02 cm. An uncertainty in the positioning of source applicator was ±0.01 cm and the outer and inner diameters of the applicator were 1.65 cm and 1.35 cm respectively, which means the lateral movement of the source inside the applicator was about ±0.022 cm. The overall uncertainty in the measurement of source-to-chamber distance could increase up to ±0.052 cm. Thus the uncertainty in the measured value in the odd situation when all the three positioning components are in one sign will be 10, 5 and 1% at distance of 1, 2 and 5 cm from the source respectively. The large uncertainty in the measured reading for small distances was minimized by the random measurements with a large sample size. The sample size of measurement used in this study depended on the distance between the source and chamber and that varied from 10 to 25. The standard error (1 σ) in the measured non-uniformity correction factor as a function of the distance between the source and chamber (cm) is shown in Figure 2. It is observed that the error increases with decrease in the source-to-chamber distance according to inverse square law. The standard error was found to be about 3.5 and 2.5% for the 0.6 and 0.1 cc ionization chamber at a distance of 1 cm from the source respectively.

Our main objective is to use these small ionization chambers, particularly 0.1 cc, for the dose measurement at shorter distances from the brachytherapy source in water medium. The important correction factor to be applied for accurate dose measurement is the non-uniformity of photon fluence. The non-uniformity correction factor in water medium is not very significantly different from that of air medium,[11] which has been discussed in the previous section. The IAEA report[10] has recommended the use of 0.6 cc ionization chamber for the measurement of air kerma strength of the brachytherapy sources by in-air calibration. The details of the calibration procedures and different correction factors have been discussed. The non-uniformity correction factor recommended by the report for 0.6 cc chamber at 10 cm is 1.009 against the 1.011 of our measured value. When the air kerma of low activity source is measured by in-air calibration, these chambers can be used for accurate measurement at shorter distances by applying the non-uniformity correction factors.

Tölli and Johansson[11] adopted the procedure of measurement of absorbed dose *D_w_* for external high-energy photon beams given by IAEA (TRS-277). They modified the IAEA formalism by introducing two correction factors to measure the absorbed dose in the center of the chamber in the high dose gradient region near the brachytherapy source. These correction factors were non-uniformity correction factor *K_n_* to account for the non-uniformity of the absorbed dose over the active volume of the chamber and the displacement correction factor *P_d_* that corrects for the difference in absorbed dose between the effective point of measurement and the center of the chamber. The modified formula for the dose measurement near the brachytherapy source based on the new protocol of IAEA (TRS-398) dose measurement is given by Equation (12).

(12) $$D_{w}\left(P_{\mathrm{center}}\right)\;=\;N_{D,w}\;M_{u}P_{d}K_{n}N_{Q}$$

where *N_D,w_* is the calibration factor of absorbed dose to water, *M_u_* is the measured reading corrected for the temperature and pressure and *P_d_* is the displacement correction factor. The displacement factor is given by *P_d_* = 1- dr, where *r* is the radius of the ionization chamber. The value of d determined for the ^192^Ir photon beams was 5.4% cm^2^ g^-1^.[14] *K_n_* is the non-uniformity correction factor. *N_Q_* is the beam quality correction factor.

## Conclusion

The experimentally derived non-uniformity correction factors were in very good agreement with the anisotropic theoretically calculated factors for both the chambers. The non-uniformity correction factors will be very much useful for an accurate dose measurement near the brachytherapy sources in the water medium using small cylindrical chambers.
